# Prognostic immune markers in esophageal cancer patients managed with trimodal therapy

**DOI:** 10.1007/s00262-024-03891-3

**Published:** 2025-01-03

**Authors:** Mark K. Farrugia, Elizabeth A. Repasky, Minhui Chen, Kristopher Attwood, Kayla Catalfamo, Hanna Rosenheck, Song Yao, David M. Mattson, Sarbajit Mukherjee, Moshim Kukar, Agnieszka K. Witkiewicz, Anurag K. Singh

**Affiliations:** 1 Departments of Radiation Medicine, Roswell Park Comprehensive Cancer Center, Elm and Carlton Streets, Buffalo, NY 14263 USA; 2 Departments of Immunology, Roswell Park Comprehensive Cancer Center, Buffalo, NY USA; 3 Department of Biostatistics and Bioinformatics, Roswell Park Comprehensive Cancer Center, Buffalo, NY USA; 4 Departments of Molecular & Cellular Biology, Roswell Park Comprehensive Cancer Center, Buffalo, NY USA; 5 Departments of Cancer Prevention & Control, Roswell Park Comprehensive Cancer Center, Buffalo, NY USA; 6 Departments of Medicine, Roswell Park Comprehensive Cancer Center, Buffalo, NY USA; 7 Departments of Surgery, Roswell Park Comprehensive Cancer Center, Buffalo, NY USA

**Keywords:** Esophageal cancer, Immunology, Prognosis, Immunofluorescence

## Abstract

**Background:**

Esophageal cancer (ESC) is an aggressive disease which often presents at an advanced stage. Despite trimodal therapy, 40–50% patients can develop metastatic disease by 18 months. Identification of patients at risk for metastatic spread is challenging with need for improved prognostication. We investigated whether the immune landscape of pretreatment tissue was associated with relapse in ESC patients.

**Methods:**

Between April 2010 and October 2018, we identified 25 patients who had undergone trimodal therapy for ESC and had pretreatment biopsies suitable for analyses. We performed high-throughput multispectral immunofluorescence (mIF) analysis on formalin-fixed paraffin embedded biopsy samples. Analysis of 27 unique populations via immune and exhaustion mIF panels was performed and expression was normalized to total cell counts.

**Results:**

Of the 25 patients analyzed, the median follow-up time was 23.9 months, during which 12 (48%) patients suffered a relapse with a median time to progression of 13.1 months. mIF revealed higher expression of HLA-DR (*p* = 0.019), CD8/LAG3 (*p* = 0.046), and CD8/CTLA4 (*p* = 0.027) among patients without relapse. Time to progression (TTP) and disease-specific survival (DSS) were stratified by median expression of each significant subpopulation and formally tested by the log-rank test. Higher than median expression of HLA-DR (*p* = 0.027), CD8/LAG3 (*p* = 0.039), and CD8/CTLA4 (*p* = 0.039) were significantly associated with TTP. Similarly, HLA-DR (*p* = 0.0069) and CD8/CTLA4 (*p* = 0.036) were significantly associated with improved DSS, whereas no significant observations were found with CD8/LAG3 (*p* = 0.11) expression. Stromal, but not tumoral expression of CD163 and CD163/PDL1 were significantly associated with improved TTP and DSS.

**Conclusions:**

High expression of HLA-DR, CD8/CTLA4, and stromal expression of CD163 and CD163/PDL1 within pretreatment biopsy ESC samples was associated with significantly reduced rates of relapse. Increased presence of these markers suggests that an improved immune landscape is associated with less aggressive disease and may provide an opportunity for risk-based treatment strategies.

**Supplementary Information:**

The online version contains supplementary material available at 10.1007/s00262-024-03891-3.

## Introduction

Esophageal cancer (ESC) is an aggressive disease which often presents at an advanced stage. Treatment of locally advanced esophageal cancer typically consists of neoadjuvant chemoradiation (CRT) and surgery in patients who are eligible [1]. However, even in individuals who can tolerate maximal therapy for locally advanced ESC, the 2-year progression free survival (PFS) of ESC patients is only approximately 50% [2]. As such, there is a need for better understanding and treatment of ESC.

In recent years, there has been strong interest in harnessing the power of host immunity in treating cancer and controlling disease progression, motivated by the success of immune checkpoint inhibitors in prolonging patient survival in multiple other cancer types [2, 3]. Recently, adjuvant immunotherapy was demonstrated to improve progression free survival in ESC patients who did not have a complete pathologic response at surgery [2]. Interestingly, several studies also showed that the composition and quality of immune infiltrates were predictive of CRT in either the neoadjuvant or adjuvant setting, highlighting the potential of tumor immune markers for risk stratification and guidance of treatment [3].

To evaluate the clinical significance of the immune infiltrates in the tumor microenvironment of ESC, we performed multispectral immunofluorescence (mIF) by Vectra analysis on pretreatment biopsy samples. mIF allows for simultaneous assessment of multiple protein markers in fixed paraffin embedded tissues with spatial resolution. This project strove to identify relevant immune cell subsets which were correlated with treatment response and outcome in ESC.

## Methods

This project was performed under the approval of the Roswell Park Comprehensive Cancer Center Institutional Review Board (BDR-150121). We retrospectively identified 297 patients who underwent CRT for esophageal cancer at our institution between April 2010 and October 2018 [4]. Among these patients, 145 underwent esophagectomy following CRT. Of these 145 patients, 36 had a pretreatment biopsy performed at our institution (Supplemental Fig. 1). Samples were assessed for suitability for Vectra analysis and ultimately 25 patients were included.

### Patients

All patients were evaluated by at least endoscopy and positron emission tomography (PET) imaging. Patients who had clinical stage II–III carcinoma of the esophagus or gastroesophageal junction were included. Patients underwent neoadjuvant CRT followed by esophagectomy. Radiation was delivered by volumetric modulated arc therapy (VMAT) to a dose of 50.4 Gray (Gy) in 1.8 Gy fractions. The most common concurrent chemotherapy regimen was carboplatin and taxol however 5-flurouracil, oxaplatin, and leucovorin was also allowed per national guidelines [1]. All patients underwent repeat PET staging following completion of CRT and those without evidence of metastatic spread proceeded to esophagectomy. Patients who were found to have metastatic spread at surgery, such as peritoneal implants or liver lesions, were excluded from analyses. Patients were followed every three to six months with computed tomography (CT) chest, abdomen, and pelvis and endoscopic evaluation as needed. Adjuvant therapy such immunotherapy was not given in this cohort.

### mIF staining

Formalin-fixed Paraffin-embedded (FFPE) 4 µm sections were cut and placed on charged slides. Slides were dried at 65 °C for 2 h. After drying, the slides were placed on the BOND RXm Research Stainer (Leica Biosystems) and deparaffinized with BOND Dewax solution (Lecia Biosystems). The mIF staining process involved serial repetitions of the following for each biomarker: epitope retrieval/stripping with ER1 (Leica Biosystems) or ER2 (Leica Biosystems), blocking buffer (AKOYA Biosciences), primary antibody, Opal Polymer HRP secondary antibody (AKOYA Biosciences), Opal Fluorophore (AKOYA Biosciences). All AKOYA reagents used for mIF staining come as a kit (NEL821001KT). Spectral DAPI (AKOYA Biosciences) was applied once slides were removed from the BOND. They were cover slipped using an aqueous method and Diamond antifade mounting medium (Invitrogen ThermoFisher). The mIF panel consisted of the following antibodies:

Panel 1 (Exhaustion): CD86 (Cell Signaling, #91882, Opal 480), TIM-3 (Cell Signaling, #45208, Opal 520), LAG-3 (Abcam, #ab209236, Opal 540), CD4 (Leica Biosystems, #NCL-L-CD4-368, Opal 570), HLA-DR (ThermoFisher, #MA5-32232, Opal 620), CTLA-4 (BioCare, #3211, Opal 650), CD8 (Agilent Dako, #M7103, Opal 690), CD80 (Abcam, #ab134120, Opal 780).

Panel 2 (Immune): Cytokeratin AE1/AE3 (Agilent Dako, #M3515, Opal 480), CD3 (Abcam, #ab16669, Opal 520), FOXP3 (Abcam, #ab20034, Opal 540), CD8 (Agilent Dako, #M7103, Opal 570), OX40 (Cell Signaling, #61637, Opal 620), PD-L1 (Cell Signaling, #13684, Opal 650), PD-1 (Cell Signaling, #43248, Opal 690) CD163 (Leica Biosystems, #NCL-L-CD163, Opal 780)Tissue Imaging and analysis.

Slides were imaged on the PhenoImager™ HT (AKOYA Biosciences). Further analysis of the slides was performed using inForm® Software v2.6.0 (AKOYA Biosciences). The RStudio plugin, phenoptrReports, was used to extract phenotype counts from the resulting data tables. Expression of Cytokeratin AE1/AE3 was used delineate tumor versus stroma. Co-expression of multiple markers is denoted by “/”. For example, CD8+/CTLA+ corresponds to tissue which co-expressed CD8 and CTLA4.

### Survival analysis

Differential expression by relapse and pathologic response was assessed by the Mann Whitney U test. Time to progression (TTP) and disease-specific survival (DSS) were estimated using the Kaplan Meier method. For TTP, progression was determined by biopsy or radiographic progression and patients who were lost to follow-up or died without documented recurrence were censored. For DSS, patients who died with a history of a documented recurrence were termed an event and others were censored. Timeframes were calculated from the start of chemoradiation to the date of progression, death, or last follow-up. Statistical Analysis was performed using R (Version 4.0.2, R Project for Statistical Computing, Vienna, Austria).

## Results

### Patient demographics

Within this cohort, the median age was 68 years (interquartile range (IQR) 58–70.5 years), 23 (92.0%) were male, 21 (84.0%) had adenocarcinoma, and 23 (92.0%) had a Karnofksy Performance Scale of at least 80 (Table 1). Clinical T3 disease (92.0%) and nodal involvement (80.0%) were evident in most patients at presentation. Pathologic complete response (pCR) to neoadjuvant CRT occurred in 5 (20.0%) of patients. During the follow-up period (median 23.9 months, IQR 15–38.8 months), 12 patients (48.0%) had disease recurrence; all were distant. The median TTP and DSS was 13.1 months and 23.9 months respectively. At the time of analysis, all patients within this cohort had died.

**Table 1:** Patient Demographics

	Overall	
	*n* = 25	
Age	Mean (SD)	65.24 (9.24)
	Median (IQR)	68.0 (58.0, 70.5)
Sex	Male	23 (92.0%)
	Female	2 (8.0%)
KPS	70	2 (8.0%)
	80	20 (80.0%)
	90	3 (12.0%)
Pathology	Adeno	21 (84.0%)
	SCC	4 (16.0%)
Tobacco use	Never	8 (32.0%)
	Former	10 (40.0%)
	Current	7 (28.0%)
T-Stage	2	1 (4.0%)
	3	23 (92.0%)
	4	1 (4.0%)
N-Stage	0	5 (20.0%)
	1+	20 (80.0%)
ypT Stage	0	5 (20.0%)
	1	7 (28.0%)
	2	3 (12.0%)
	3	10 (40.0%)
ypN Stage	0	16 (64.0%)
	1+	9 (36.0%)
pCR	Yes	5 (20.0%)
	No	20 (80.0%)
Relapse	No	13 (52.0%)
	Yes	12 (48.0%)
TTP	Mean (SD)	21.7 (20.7)
	Median (IQR)	13.1 (8.9, 22.4)
DSS	Mean (SD)	33.3 (28.3)
	Median (IQR)	23.9 (15, 40.6)

### Vectra analysis

Vectra results for the immune and exhaustion panels are shown in Table 2. Notably, data for the immune and exhaustion panels were unable to be obtained for 1 and 2 unique patients respectively. Of these markers, human leukocyte antigen-DR isotype (HLA-DR)+, CD8+/lymphocyte activating gene 3 (LAG3) + , CD8 + / cytotoxic T-lymphocyte associated protein 4 (CTLA4)+, and CD163+/programmed death-ligand 1 (PDL1)+ demonstrated significant enrichment in patients who did not relapse. Of the evaluated populations, AE1AE3+/PDL1+ (*p* = 0.046) levels were elevated in patients who went on to have pCR to neoadjuvant CRT however these markers did not correlate with relapse (Supplemental Table 1). Via pan-cytokeratin expression, the stromal and tumoral expression of the immune panel markers we examined. As the exhaustion panel lacked this marker, unfortunately this analysis could not be performed for these markers. Stromal expression of CD163+, CD163+/PDL1+, and CD3+/CD8−/PDL1− were significantly enriched in those without relapse. There was no significant relationship in the tumoral expression of any of the examined subpopulations and relapse (Table 3).

**Table 2:** mIF expression in those with or without relapse

		Overall	Relapse	Non-Relapse	*p*-value
		*n* = 25	*n* = 12	*n* = 13	
CD4+	Mean	7.557	5.151	9.763	0.1693
	Median (IQR)	3.778 (1.913, 9.621)	2.464 (1.57, 4.435)	6.468 (2.569, 13.27)	
CD8+	Mean	3.749	2.874	4.551	0.2875
	Median (IQR)	2.215 (1.293, 4.691)	1.585 (1.145, 2.437)	2.9 (1.332, 5.164)	
HLA-DR+	Mean	3.118	1.997	4.146	**0.01879**
	Median (IQR)	2.161 (1.662, 3.782)	1.773 (0.9848, 1.998)	2.577 (2.258, 6.615)	
CD4+/LAG3+	Mean	0.07583	0.02432	0.1231	0.09084
	Median (IQR)	0.0123 (0.007816, 0.05724)	0.008988 (0.006588, 0.0273)	0.02797 (0.009903, 0.08872)	
CD4+/TIM3+	Mean	0.8083	0.1865	1.378	0.2066
	Median (IQR)	0.06002 (0.01803, 0.3802)	0.02762 (0.005027, 0.2664)	0.099 (0.02491, 1.403)	
CD4+/CTLA4+	Mean	0.6882	0.2742	1.068	0.09084
	Median (IQR)	0.1474 (0.05249, 0.8108)	0.1107 (0.03678, 0.2445)	0.5096 (0.06714, 1.256)	
CD8+/LAG3+	Mean	0.01816	0.002834	0.0322	**0.04562**
	Median (IQR)	0.004091 (0.0, 0.01044)	0.0 (0.0, 0.004306)	0.006307 (0.003068, 0.02984)	
CD8+/TIM3+	Mean	0.2934	0.04615	0.52	0.06916
	Median (IQR)	0.03451 (0.008065, 0.1397)	0.01151 (0.001134, 0.0899)	0.06367 (0.02154, 0.3269)	
CD8+/CTLA4+	Mean	0.04173	0.01874	0.0628	**0.0268**
	Median (IQR)	0.02152 (0.007816, 0.05048)	0.01111 (0.004586, 0.02215)	0.03316 (0.01754, 0.1023)	
CD80+/CD86−	Mean	5.058	3.226	6.737	0.1335
	Median (IQR)	1.402 (0.2707, 9.38)	0.7898 (0.2197, 2.193)	3.403 (0.8115, 10.34)	
CD80−/CD86+	Mean	0.7036	0.5207	0.8712	0.5254
	Median (IQR)	0.2905 (0.2037, 0.5461)	0.2256 (0.2117, 0.3466)	0.364 (0.1786, 0.6784)	
CD80+/CD86+	Mean	0.741	0.2045	1.233	0.4134
	Median (IQR)	0.1905 (0.03752, 0.5204)	0.1322 (0.03216, 0.2865)	0.2298 (0.0814, 1.265)	
HLA-DR+/CD4+	Mean	1.411	0.718	2.047	0.0595
	Median (IQR)	0.542 (0.351, 1.758)	0.3982 (0.163, 0.5645)	0.9482 (0.4754, 2.687)	
HLA-DR+/CD8+	Mean	0.2201	0.1466	0.2876	0.05122
	Median (IQR)	0.09003 (0.05048, 0.2024)	0.06292 (0.0446, 0.0869)	0.1838 (0.089, 0.3906)	
HLA-DR+/CD80+/CD86−	Mean	0.7158	0.3075	1.09	0.03174
	Median (IQR)	0.1757 (0.03451, 0.7881)	0.05216 (0.02113, 0.2108)	0.5707 (0.09231, 1.568)	
HLA-DR+/CD80−/CD86+	Mean	0.256	0.2313	0.2787	0.347
	Median (IQR)	0.09787 (0.05648, 0.1854)	0.06333 (0.05326, 0.1211)	0.1466 (0.06633, 0.1994)	
HLA-DR+/CD80+/CD86+	Mean	0.3756	0.1123	0.617	0.2875
	Median (IQR)	0.09222 (0.02914, 0.2707)	0.08887 (0.028, 0.1297)	0.1764 (0.04402, 0.6234)	
CD163+	Mean	7.482	4.005	10.96	0.05966
	Median (IQR)	5.169 (3.137, 8.156)	3.569 (2.728, 5.667)	7.34 (4.333, 12.83)	
CD8+	Mean	3.646	2.489	4.803	0.3474
	Median (IQR)	1.378 (0.7702, 3.492)	1.258 (0.4906, 2.184)	2.267 (1.0, 5.053)	
AE1AE3+/PDL1+	Mean	0.2034	0.1165	0.2903	0.2598
	Median (IQR)	0.04483 (0.01983, 0.2662)	0.03369 (0.01851, 0.0656)	0.1222 (0.03103, 0.3862)	
CD8+/PD1+	Mean	0.8015	0.5548	1.048	0.2913
	Median (IQR)	0.2026 (0.03479, 0.4775)	0.09116 (0.02376, 0.7222)	0.3038 (0.1357, 0.3924)	
CD8+/PDL1+	Mean	0.1801	0.06587	0.2944	0.2566
	Median (IQR)	0.01336 (0.001555, 0.07025)	0.01004 (0.0, 0.02125)	0.0202 (0.006638, 0.3536)	
CD8+/OX40+	Mean	0.09979	0.04815	0.1514	0.2986
	Median (IQR)	0.04079 (0.01148, 0.07299)	0.02548 (0.008768, 0.06369)	0.04399 (0.02908, 0.1635)	
CD3+/CD8−	Mean	12.03	9.907	14.16	0.2913
	Median (IQR)	7.225 (4.594, 17.08)	5.283 (4.091, 14.4)	11.73 (6.293, 20.9)	
CD3+/CD8−/PD1+	Mean	1.026	0.6465	1.405	0.7987
	Median (IQR)	0.4559 (0.153, 1.052)	0.4059 (0.1043, 0.7886)	0.4599 (0.2506, 1.405)	
CD3+/CD8−/PDL1+	Mean	0.4541	0.2163	0.6918	0.2365
	Median (IQR)	0.1293 (0.03987, 0.3365)	0.1031 (0.01411, 0.2043)	0.1634 (0.05101, 1.123)	
CD3+/CD8−/FOXP3+	Mean	0.5134	0.3922	0.6347	0.9323
	Median (IQR)	0.3417 (0.2031, 0.6079)	0.2863 (0.2031, 0.6206)	0.4028 (0.2138, 0.5102)	
CD163+/PDL1+	Mean	0.4817	0.1352	0.8282	**0.0449**
	Median (IQR)	0.08384 (0.02639, 0.4696)	0.052 (0.01337, 0.123)	0.2501 (0.08003, 1.19)	

Bold indicates *p* < 0.05

**Table 3:** Stromal and tumoral mIF expression in those with or without relapse

		Overall	Relapse	Non-Relapse	*p*-value
		Median (IQR), *n* = 25	Median (IQR), *n* = 12	Median (IQR), *n* = 13	
CD8+	Stroma	1.142 (0.886–6.583)	1.494 (0.813–5.238)	1.42 (1.13–8.14)	0.437
	Tumor	1.709 (0.882–3.18)	1.378 (0.582–2.504)	2.789 (1.033–3.404)	0.205
AE1AE3+/PDL1+	Stroma	0.091 (0.009–0.278)	0.0691 (0.006–0.100)	0.163 (0.035–0.293)	0.265
	Tumor	0.028 (0.003–0.274)	0.014 (0.002–0.279)	0.131 (0.007–0.231)	0.461
CD8+/PD1+	Stroma	0.258 (0.039–0.739)	0.174 (0.019–1.512)	0.258 (0.076–0.538)	0.605
	Tumor	0.285 (0.014–0.802)	0.098 (0.018–0.624)	0.369 (0.015–0.851)	0.683
CD8+/PDL1+	Stroma	0.008 (0–0.23)	0.004 (0–0.071)	0.010 (0–0.895)	0.249
	Tumor	0.0183 (0.004–0.062)	0.011 (0–0.028)	0.023 (0.008–0.087)	0.163
CD8+/OX40+	Stroma	0.029 (0–0.107)	0.024 (0–0.058)	0.041 (0.025–0.225)	0.23
	Tumor	0.032 (0.007–0.108)	0.015 (0.006–0.060)	0.049 (0.022–00.233)	0.288
CD3+/CD8−	Stroma	5.65 (1.948–15.484)	4.093 (1.695–10.261)	11.431 (2.63–15.5)	0.376
	Tumor	6.767 (3.338–11.775)	6.699 (3.692–7.444)	7.791 (3.269–11.941)	0.611
CD3+/CD8−/PD1+	Stroma	0.296 (0.082–1.122)	0.546 (0.041–1.453)	0.288 (0.165–0.497)	0.892
	Tumor	0.267 (0.105–0.802)	0.247 (0.138–0.495)	0.579 (0.088–1.776)	0.437
CD3+/CD8−/PDL1+	Stroma	0.073 (0.015–0.756)	0.014 (0–0.28)	0.082 (0.057–2.443)	0.047
	Tumor	0.131 (0.046–0.219)	0.099 (0.018–0.148)	0.158 (0.11–0.27)	0.2
CD3+/CD8−/FOXP3 +	Stroma	0.225 (0.098–0.691)	0.214 (0.108–0.666)	0.251 (0.089–1.149)	0.979
	Tumor	0.28 (0.121–0.533)	0.272 (0.168–0.381)	0.347 (0.080–0.691)	0.769
CD163+/PDL1+	Stroma	0.129 (0.010–0.489)	0.015 (0–0.138)	0.406 (0.113–1.788)	0.011
	Tumor	0.069 (0.022–0.218)	0.053 (0.153–0.08)	0.127 (0.044–0.621)	0.1095
CD163+	Stroma	3.4 (0.009–7.786)	2.336 (1.650–3.473)	7.28 (2.545–14.084)	0.039
	Tumor	3.68 (2.673–6.829)	3.213 (2.418–4.718)	5.709 (3.603–9.244)	0.052

Subgroup analysis restricted to patients with adenocarcinoma or those that did not have a complete response to CRT yielded similar results with the notable exception being enrichment of HLA-DR/CD80+/CD86+ expression in patients without a history of relapse (Supplemental Table 2). Additional subgroup analysis included omitting patients that had a complete pathologic response to treatment which also yielded similar results (Supplemental Table 3).

### Time to progression

**Fig. 1:**
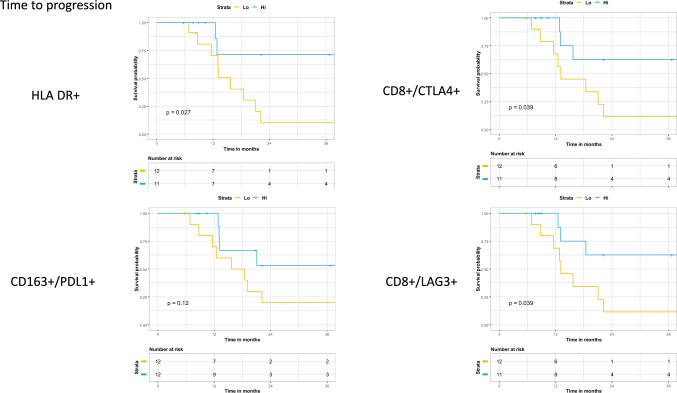
Time to progression stratified by the median expression of each respective population via the Kaplan Meier method

To further characterize the relationship between the significant populations and relapse, TTP and DSS were stratified by high vs. low expression based on median expression levels. High expression of HLA-DR+ (*p* = 0.027), CD8+/LAG3+ (*p* = 0.039), CD8+/CTLA+ (*p* = 0.039), but not CD163+/PDL1+ (*p* = 0.12) were significantly associated with TTP (Fig. 1). Omission of squamous cell carcinoma (SCC) patients or those who had a pCR did not significantly change these observations (Supplemental Figs. 2–3). While, HLA-DR/CD80+/CD86+ was significantly enriched in those without relapse in both subgroups, HLA-DR/CD80+/CD86+ expression only trended with improved TTP (*p* = 0.07) in those who did not have a pCR to CRT.

**Fig. 2:**
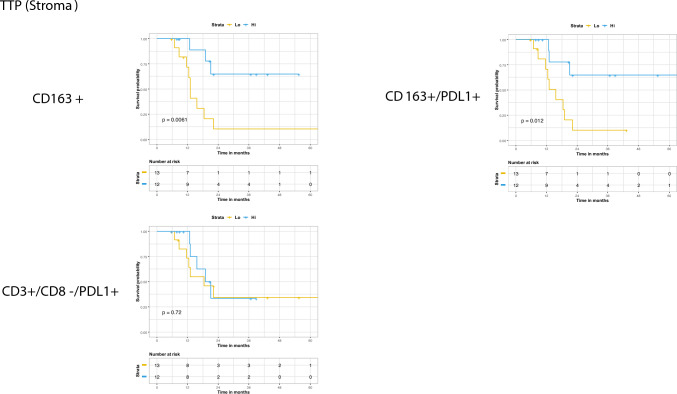
Time to progression stratified by the median stromal expression of each respective population via the Kaplan Meier method

Within the stromal compartment, higher than median expression of CD163+ (*p* = 0.0061) and CD163+/PDL1+ (*p* = 0.012) but not CD3+/CD8−/PDL1+ was associated with improved TPP (Fig. 2). The median tumoral expression of CD163+ or CD163+/PDL1+ was not associated with outcome in this cohort (Supplemental Fig. 4).

### Disease-specific survival

**Fig. 3:**
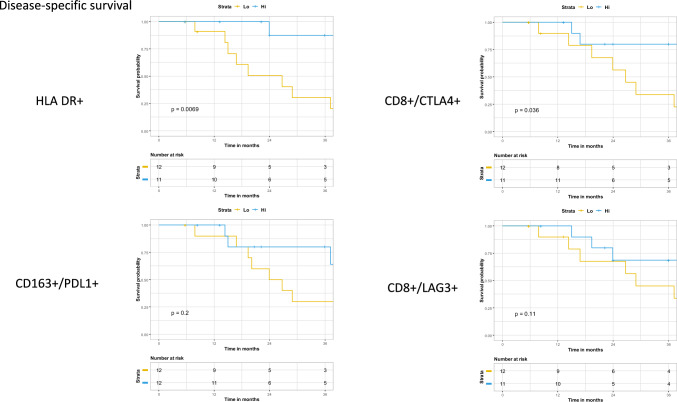
Disease specific survival stratified by the median expression of each respective population Kaplan Meier method

Similarly, high expression of HLA-DR+ (*p* = 0.0069) and CD8+/CTLA4+ (*p* = 0.036) were significantly associated with improved DSS, whereas no significant observations were found with CD8+/LAG3+ (*p* = 0.11) or CD163+/PDL1+ (*p* = 0.2) staining (Fig. 3). When evaluating only adenocarcinoma samples, only HLA-DR+ and CD8+/CTLA4+ expression remained significantly correlated with DSS (Supplemental Fig. 2). Interestingly, high expression of HLA-DR+, CD8+/CTLA4+, or HLA-DR/CD80+/CD86+ were associated with improved DSS in patients without a pCR to CRT (Supplemental Fig. 3).

**Fig. 4:**
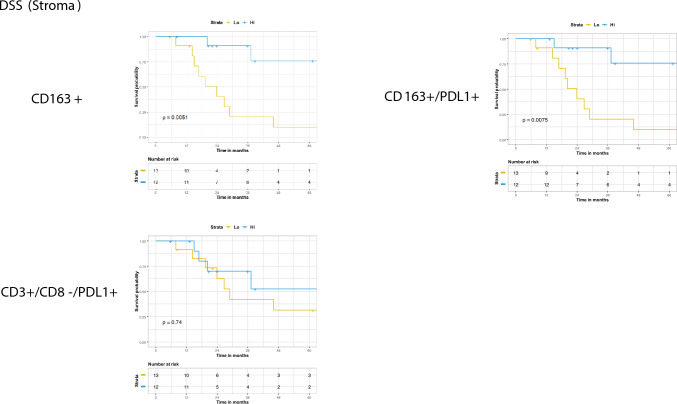
Disease specific survival stratified by the median stromal expression of each respective population Kaplan Meier method

Within the stromal compartment, higher than median expression of CD163+ (*p* = 0.0051) and CD163+/PDL1+ (*p* = 0.0075) but was associated with improved DSS (Fig. 4). As with TTP, tumoral expression of CD163+ was not associated with DSS (Supplemental Fig. 4.)

**Fig. 5:**
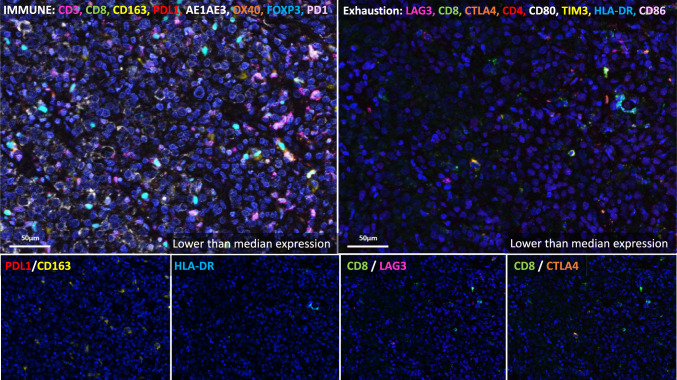
Multispectral immunofluorescence (mIF) using immune (left) and exhaustion (right) marker panels in a representative patient who experienced early metastatic spread. The bottom panels correspond to mIF for each respective cell population within this patient. For each of the represented cell populations (PDL1/CD163, HLA-DR, CD8/LAG3, CD8/CTLA4), expression was below median expression in this patient

**Fig. 6:**
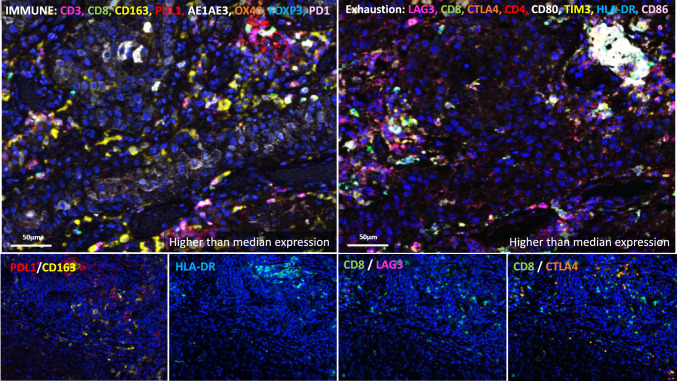
Multispectral immunofluorescence (mIF) using immune (left) and exhaustion (right) marker panels in a representative patient who remained disease-free. The bottom panels correspond to mIF for each respective cell population within this patient. For each of the represented cell populations (PDL1/CD163, HLA-DR, CD8/LAG3, CD8/CTLA4), expression was above median expression in this patient

Representative mIF for a patient with low expression of HLA-DR, CD8+/LAG3+, CD8+/CTLA4+, CD163+/PDL1+ and exhibited early metastases as well as a patient with high expression of each marker without relapse is shown in Figs. 5 and 6 respectively.

## Discussion

In this report, Vectra analysis identified several enriched immune populations in the pretreatment biopsy samples of esophageal cancer patients who did not suffer a relapse. Further characterization found stromal expression of CD163, along with HLA-DR and CD8/CTLA4 positivity to significantly stratify both TTP and DSS, potentially identifying novel markers which could be used for risk-adapted care.

RNAseq analysis has highlighted the diverse immune tumor microenvironment (TME) of gastroesophageal cancer [5]. Derks et al. demonstrated that more proximally located tumors, as well as patients from North America and Europe exhibit a greater degree of immune infiltration within the tumor microenvironment [5]. The immune-inflamed TME or tumoral T-cell infiltration has been associated with superior outcome when compared to an immunosuppressive TME [6–9]. For example, tumoral CD163+ enrichment has been associated with poor response to treatment and shortened disease-free survival [7–9]. While, no unfavorable relationships between tumoral CD163 expression and outcome were observed in the current study, stromal enrichment of CD163+ and CD163+/PDL1+ cells was significantly associated with prolonged TTP and DSS. These observations highlight the significance of compartmental expression of immune markers and patient outcomes.

The expression of HLA-DR in tumor epithelium is a previously reported independent prognostic indicator in ESC patients [10]. Increased expression of HLA-DR in tumors has been reported with favorable outcomes in multiple cancer histologies [11–13]. Others have found high levels of HLA-DR+ cells in peripheral blood to correlate with pCR following neoadjuvant CRT [14]. Consistent with these reports, high HLA-DR expression associated with improved TTP and DSS was demonstrated in the present study. Unfortunately, we were unable to delineate whether tumor vs stromal expression was most correlated with improved outcome. However, given that Dunne et al. found tumor expression of HLA-DR to be prognostically favorable, we propose that upregulated expression of HLA-DR by tumor epithelium potentially process and present antigens to T cells, playing a direct role in antitumor immunity [10].

High expression of CD8+/CTLA-4+ was associated with an independent positive outcome in this ESC cohort. Upon activation, CTLA-4 in T cells is normally upregulated [15]. Thus, high CTLA-4 expression in this setting presumptively reflects a tumor microenvironment with high infiltration of activated immune cells, as opposed to CTLA-4 expressing regulatory T cells and exhausted cytotoxic T cells [16]. The positive associations between CD8+/CTLA-4+ and outcomes, including TTP and DSS in ESC patients, may reflect a tumor microenvironment in which antitumor immunity properties dominate. In fact, co-expression of CD8 mitigated any negative prognostic implications of CTLA-4 or LAG-3 expression in esophageal squamous cell carcinoma patients [17]. A positive influence of interstitial lymphocytes with high CTLA-4 on outcome has also been reported in breast cancer patients [18].

In the current study, there was no correlation between the aforementioned significant subpopulations and pCR suggesting these findings are independent to sensitivity to CRT. In fact, only AE1AE3+/PDL1+ was significantly associated with pCR and this did not translate to associations with TTP or DSS. Supporting this, the key findings of this report were unaltered when eliminating patients who had a complete response to neoadjuvant CRT.

In the current study, the expression of select immune markers in pretreatment tissue consistent with an unfavorable immune profile was associated with development of early metastases. Patients who ultimately develop early metastases may not benefit from the improved local control of trimodal therapy yet will still incur the reduction of in quality of life associated with these therapies [19]. Therefore, the immune expression within pretreatment tissues could help guide practitioners to either local or systemic treatment intensification. For example, EA2174 (NCT03604991) is examining the benefit of adding immunotherapy the CRT and dual checkpoint inhibition in the adjuvant setting. The immune landscape may predict which patients would benefit from this approach. This is of particular interest as CD8/CTLA-4 expression was prognostic in this study and EA2174 incorporates the CTLA-4 inhibitor ipilimumab in the adjuvant setting.

Our study has several limitations. Due to referral patterns, most patients were biopsied prior to initial evaluation at our institution thereby significantly limiting the number of patients with available pretreatment tissue for analysis. Given the small sample size, we could not statistically adjust for potential confounding clinical variables. Additionally, we were unable to delineate between stromal versus tumoral expression within the exhaustion markers. Furthermore, while the main cohort included patients with SCC, they represented a small component of this study and the major conclusions were unaltered by omitting these samples. As such, it is unclear how applicable these findings are to esophageal SCC. Future prospective investigations plan on addressing this issue by incorporating additional biopsies if needed at the time of initial endoscopic evaluation at our institution. Lastly, these patients were treated prior to the results of Checkmate 577 and it is unclear if adjuvant immunotherapy would alter these findings.

Nonetheless, our reported findings could have significant clinical implications. To date, only treatment response is routinely used to direct used to adapt patient care in locally advanced, non-metastatic esophageal cancer [1]. With a complete clinical response, omission of surgery has shown to yield non-inferior rates of OS and improved quality of life (QOL) per the surgery versus active surveillance for oesophageal cancer (SANO) trial (NTR6803 8-11-2017). Additionally, pathologic response to neoadjuvant CRT guides the use of adjuvant immunotherapy following esophagectomy. However even pCR has its limitations as surgical resection is required and even those who attain a pCR have an approximately 25% chance of relapse [20, 21].

In conclusion, mIF profiling of the immune landscape in pretreatment biopsy samples of esophageal cancer identified HLA-DR and CD8/CTLA-4 positivity to be significantly associated with TTP and DSS. Specifically, stromal expression of CD163+ and CD163+/PDL1+ significantly correlated with improved outcomes. Expression of these relevant subpopulations could be useful in prognostication and treatment personalization in ESC. Future studies validating the importance of these markers is warranted.

## Supplementary Information

Below is the link to the electronic supplementary material.

Supplementary file 1 (PDF 10 kb)

Supplementary file 2 (PDF 59 kb)

Supplementary file 3 (PDF 45 kb)

Supplementary file 4 (PDF 22 kb)

Supplementary file 5 (DOCX 20 kb)

Supplementary file 6 (DOCX 23 kb)

Supplementary file 7 (DOCX 22 kb)

## Data Availability

No datasets were generated or analyzed during the current study.
