# Supplementary figures and images for: Prognostic immune markers in esophageal cancer patients managed with trimodal therapy
Source: Cancer Immunol Immunother. 2025 Jan 3;74(2):57. doi: 10.1007/s00262-024-03891-3 (PMC11698998; doi:10.1007/s00262-024-03891-3)

Eligibility

Consort Diagram

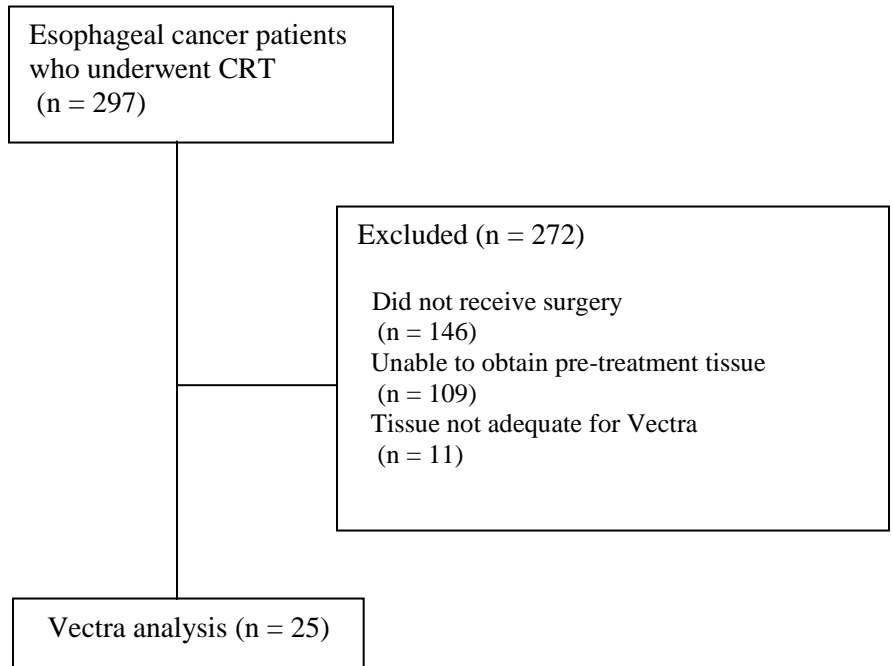

Supplement: Supplementary file 1 — (PDF 10 kb) [file 262_2024_3891_MOESM1_ESM.pdf]

CD163+

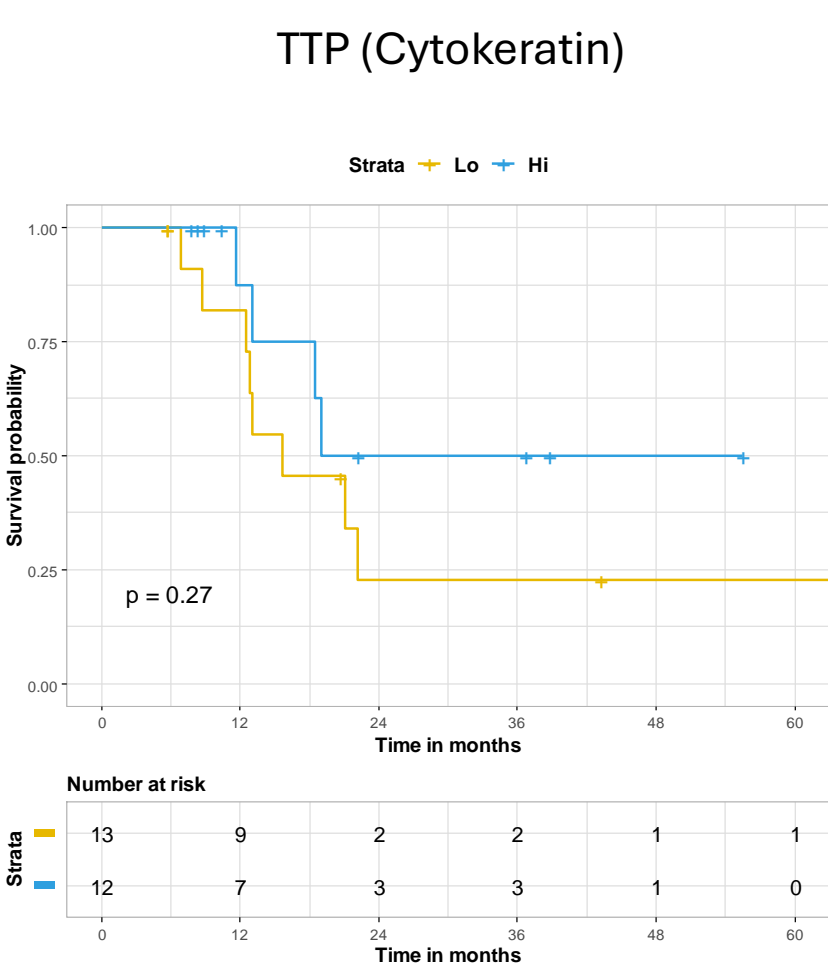

CD163+

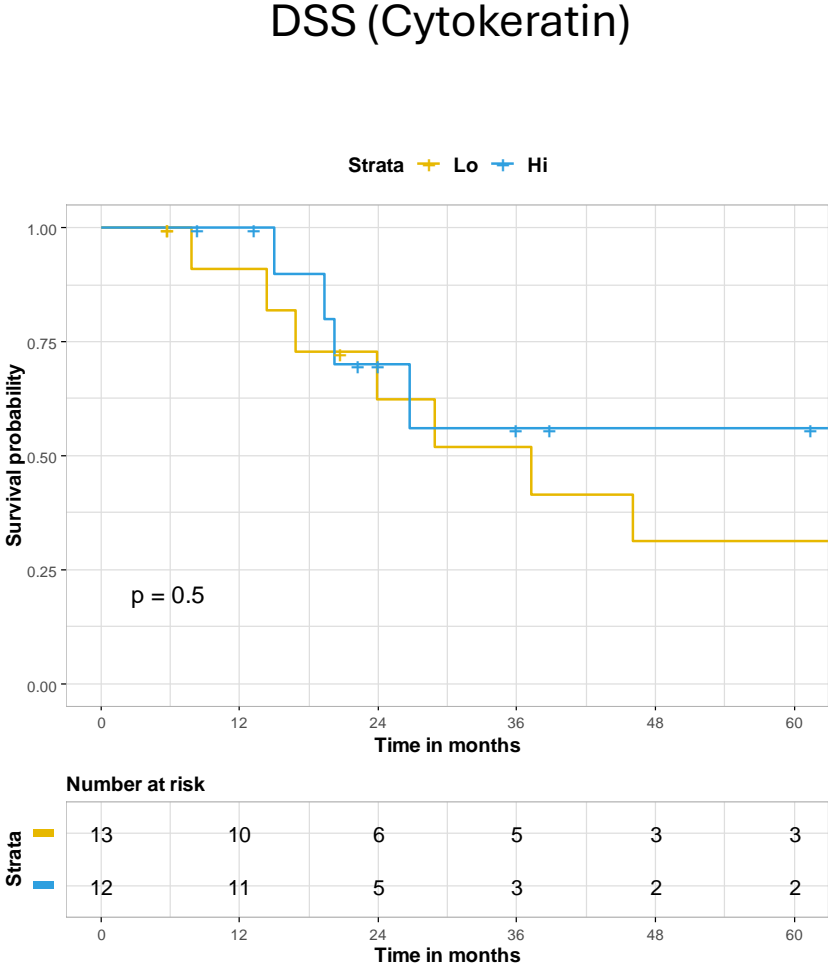

Supplement: Supplementary file 4 — (PDF 22 kb) [file 262_2024_3891_MOESM4_ESM.pdf]
