# Supplementary material for: Prognostic immune markers in esophageal cancer patients managed with trimodal therapy
Source: Cancer Immunol Immunother. 2025 Jan 3;74(2):57. doi: 10.1007/s00262-024-03891-3 (PMC11698998; doi:10.1007/s00262-024-03891-3)

TTP (Adeno only)

HLA DR+

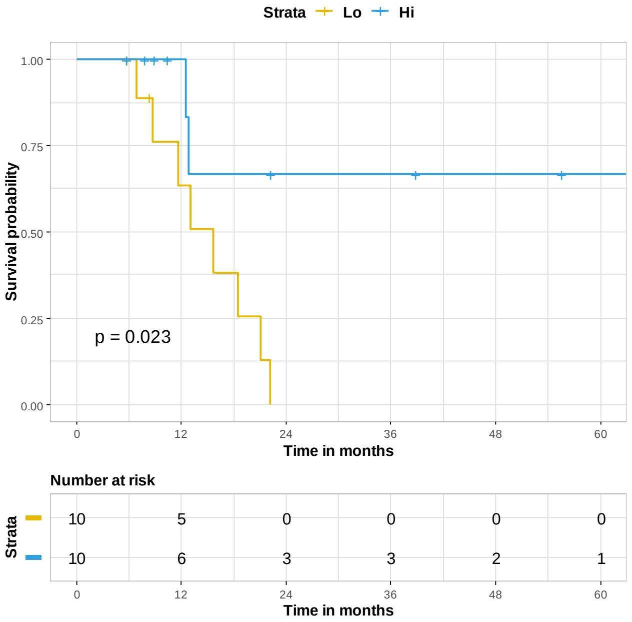

CD8+/CTLA4+

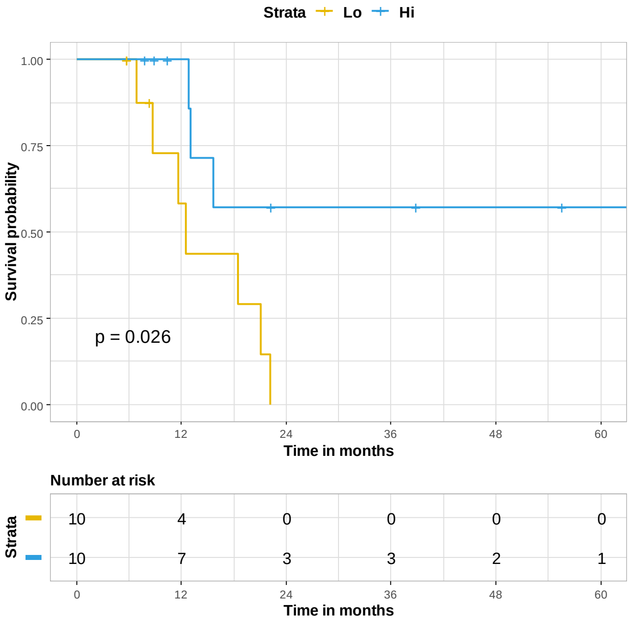

HLA-DR+/CD80+/CD86+

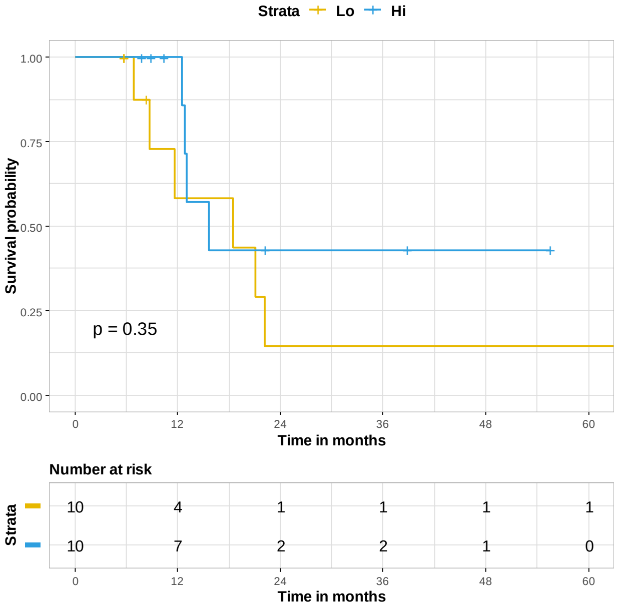

CD8+/LAG3+

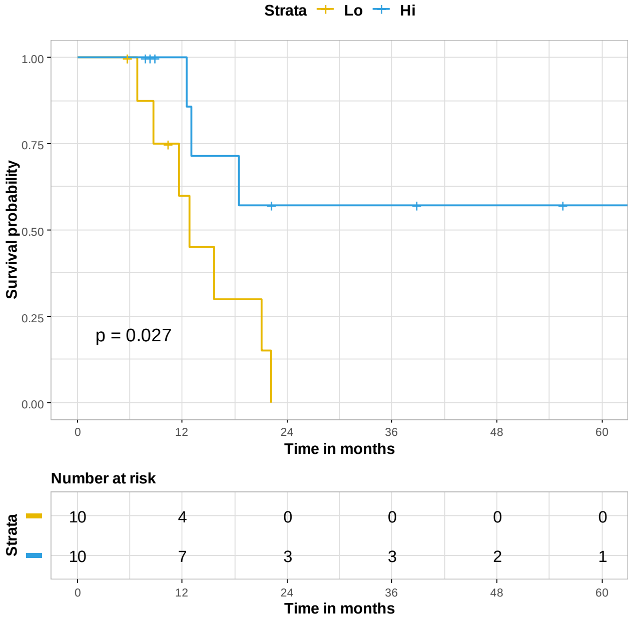

DSS (Adeno only)

HLA DR+

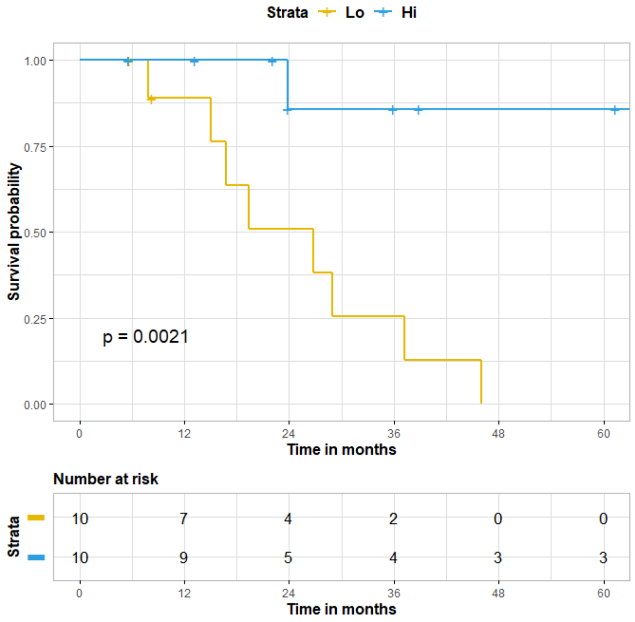

CD8+/CTLA4+

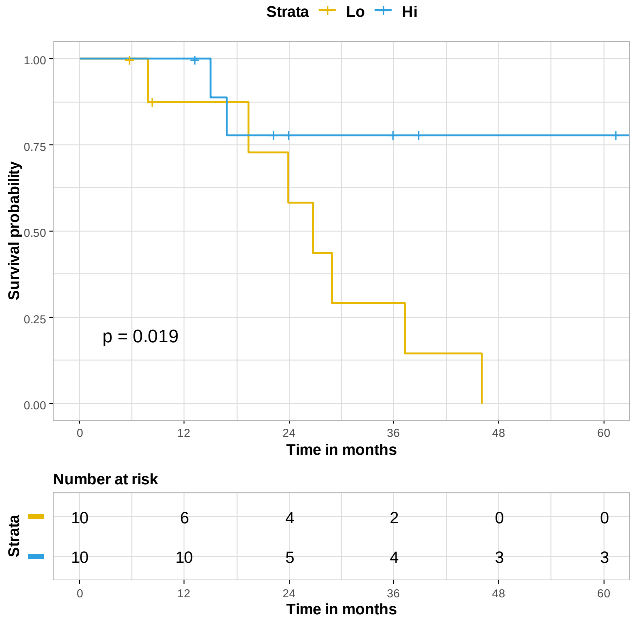

HLA-DR+/CD80+/CD86+

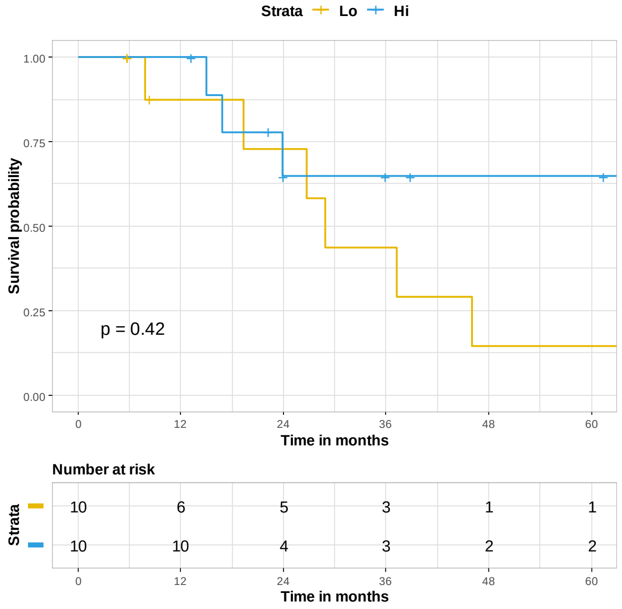

CD8+/LAG3+

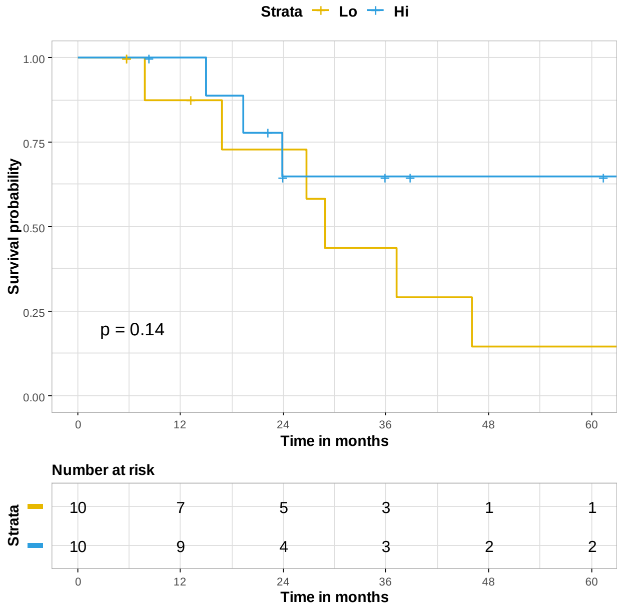

OS (Adeno only)

HLA DR+

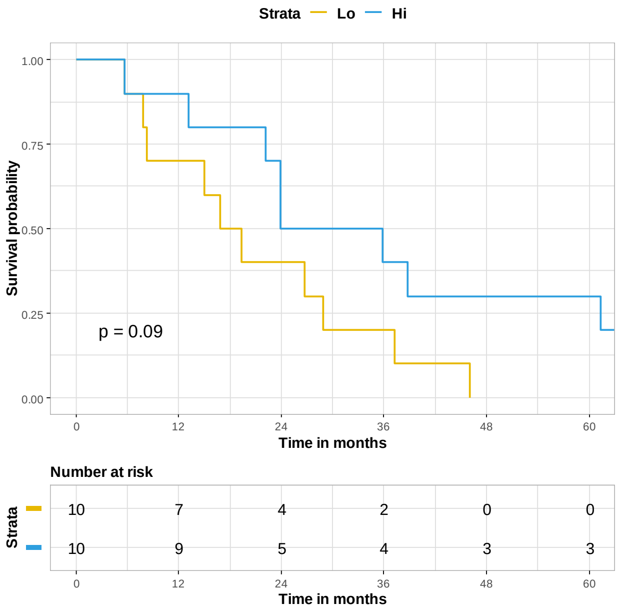

CD8+/CTLA4+

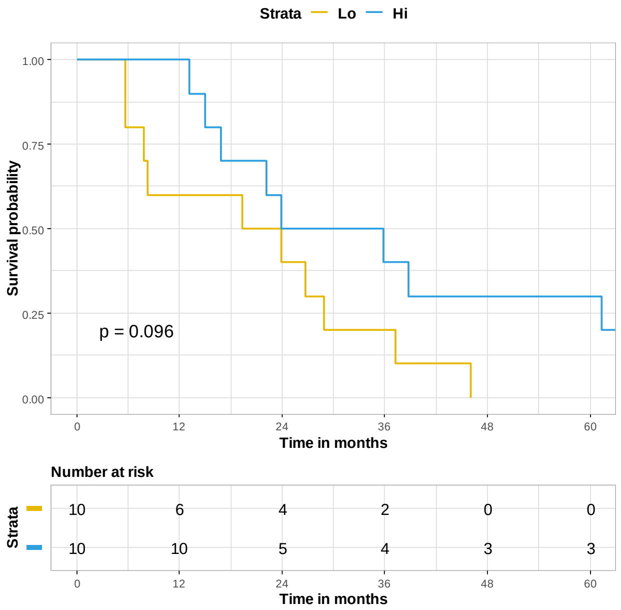

HLA-DR+/CD80+/CD86+

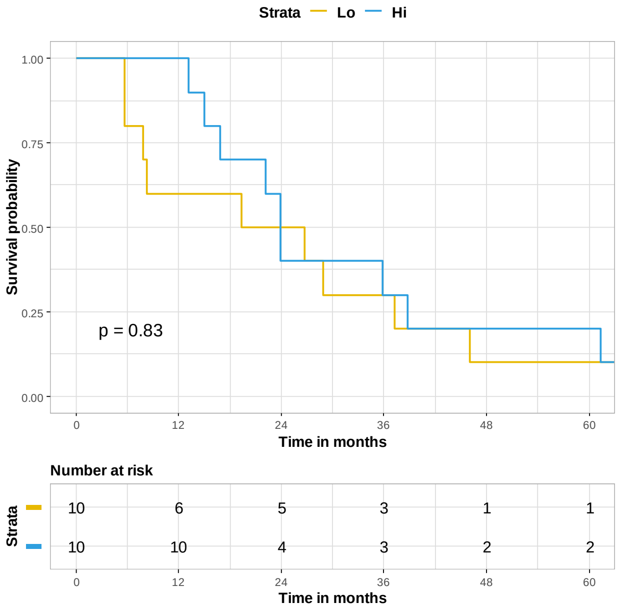

CD8+/LAG3+

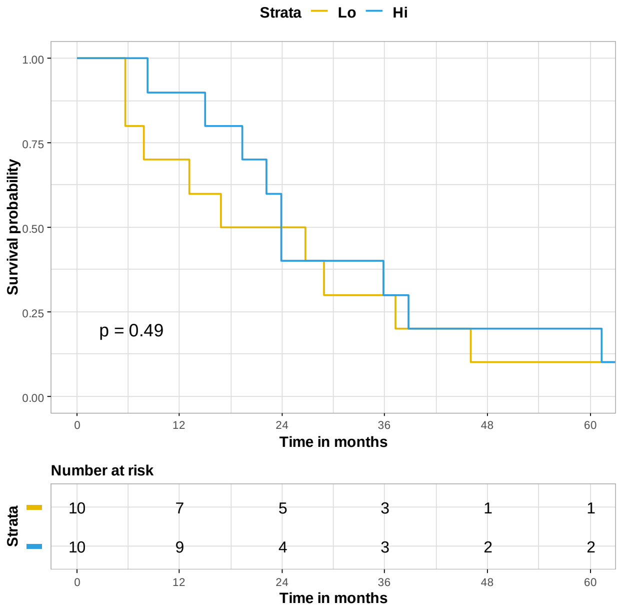

Supplement: Supplementary file 2 — (PDF 59 kb) [file 262_2024_3891_MOESM2_ESM.pdf]
