# Supplementary material for: Prognostic immune markers in esophageal cancer patients managed with trimodal therapy
Source: Cancer Immunol Immunother. 2025 Jan 3;74(2):57. doi: 10.1007/s00262-024-03891-3 (PMC11698998; doi:10.1007/s00262-024-03891-3)
Supplement: Supplementary file 4 [file 262_2024_3891_MOESM5_ESM.docx]

	pCR (n=5)			No pCR (n=20)			
	Median	Lower Quartile	Upper Quartile	Median	Lower Quartile	Upper Quartile	p-value
**CD4+**	5.058486595	4.456893102	7.878481013	3.261743379	1.771836822	14.57061078	0.74
**CD8+**	2.449880957	1.117974684	3.349750584	2.211081352	1.319150406	4.824503879	0.86
**HLA-DR+**	2.401573445	2.290005848	3.316704867	1.997992273	1.241779589	5.323148554	0.54
**CD4+/LAG3+**	0.010126582	0.009233895	0.013802146	0.019063685	0.007417005	0.065496385	1
**CD4+/TIM3+**	0.026329114	0.015389824	0.089713951	0.084152579	0.022301791	0.385324694	0.39
**CD4+/CTLA4+**	0.449620253	0.058481332	1.142127601	0.129239358	0.052378159	0.670712782	0.8
**CD8+/LAG3+**	0.01348254	0	0.024623719	0.003672399	0	0.00633846	0.46
**CD8+/TIM3+**	0.024623719	0.006075949	0.034505366	0.063674749	0.010418048	0.147886067	0.53
**CD8+/CTLA4+**	0.024623719	0.010126582	0.091557745	0.020761639	0.007633374	0.03746913	0.8
**CD80+/CD86-**	0.378734177	0.221613469	4.364928746	1.785802656	0.437240317	9.515101882	0.64
**CD80+/CD86+**	0.400262241	0.290459052	0.486318446	0.246206392	0.200007218	0.549776892	0.45
**CD80+/CD86+**	0.269141852	0.10157284	0.278639162	0.161314976	0.030983799	0.613937554	0.86
**HLA-DR+/CD4+**	0.824678238	0.495552341	0.856708861	0.482494744	0.330079605	2.24230337	0.91
**HLA-DR+/CD8+**	0.176801162	0.062918521	0.190833821	0.087965316	0.049647958	0.309211657	0.8
**HLA-DR+/CD80+/CD86-**	0.101265823	0.043091508	0.472723509	0.210817883	0.032478906	0.859068981	0.8
**HLA-DR+/CD80-/CD86+**	0.156976207	0.097872072	0.189779511	0.081729895	0.051767233	0.170991786	0.26
**HLA-DR+/CD80+/CD86+**	0.196680584	0.049247438	0.242685722	0.090543017	0.027038894	0.263603707	0.64
**CD163+**	4.7327706	3.135512536	6.805615785	5.605162274	3.320258855	8.785394248	0.68
**CD8+**	1.943300087	1.05149636	2.780069819	1.37072922	0.693430326	5.275713038	1
**AE1AE3+/PDL1+**	0.374126662	0.053951127	0.474306158	0.038030044	0.008082139	0.132299397	0.046
**CD8+/PD1+**	0.377177753	0.067613252	0.437956204	0.161627686	0.031235573	0.468101134	0.48
**CD8+/PDL1+**	0.018030201	0.015867978	0.037944493	0.010652463	0	0.099037564	0.47
**CD8+/OX40+**	0.04443034	0.043553104	0.302340897	0.037113785	0.01104705	0.064653856	0.35
**CD3+/CD8-**	7.610581093	6.840088607	8.662919717	6.64160401	4.537385667	21.17629999	0.78
**CD3+/CD8-/PD1+**	0.590489069	0.533007031	1.146465742	0.322228291	0.102354778	0.836219043	0.26
**CD3+/CD8-/PDL1+**	0.193589337	0.180302006	0.238508239	0.085670576	0.015335612	0.412600832	0.39
**CD3+/CD8-/FOXP3+**	0.436361665	0.369164403	0.469692161	0.313148463	0.198292258	0.641133698	0.89
**CD163+/PDL1+**	0.082513488	0.072588506	0.159908933	0.085166074	0.012880253	0.598887023	0.73
