# Supplementary material for: Prognostic immune markers in esophageal cancer patients managed with trimodal therapy
Source: Cancer Immunol Immunother. 2025 Jan 3;74(2):57. doi: 10.1007/s00262-024-03891-3 (PMC11698998; doi:10.1007/s00262-024-03891-3)
Supplement: Supplementary file 5 [file 262_2024_3891_MOESM6_ESM.docx]

	Overall			No relapse (n=10)			Relapse (n=11)			
	Median	1st quartile	3rd quartile	Median	1st quartile	3rd quartile	Median	1st quartile	3rd quartile	p-value
**CD4+**	3.710	1.521	14.571	4.758	1.485	22.273	2.922	0.935	7.929	0.3150
**CD8+**	2.211	1.281	5.577	3.437	1.338	5.783	1.458	0.988	3.367	0.2180
**HLA-DR+**	2.225	1.663	5.469	2.577	2.161	7.752	1.790	0.840	2.404	**0.0230**
**CD4+/LAG3+**	0.013	0.008	0.065	0.028	0.008	0.147	0.009	0.007	0.050	0.1660
**CD4+/TIM3+**	0.075	0.017	0.385	0.099	0.015	3.651	0.029	0.008	0.375	0.3450
**CD4+/CTLA4+**	0.129	0.049	0.864	0.457	0.046	1.142	0.111	0.038	0.432	0.2800
**CD8+/LAG3+**	0.005	0.000	0.012	0.006	0.002	0.025	0.001	0.000	0.006	0.0580
**CD8+/TIM3+**	0.046	0.010	0.148	0.064	0.012	0.493	0.013	0.002	0.104	0.1200
**CD8+/CTLA4+**	0.022	0.007	0.057	0.033	0.008	0.135	0.009	0.002	0.026	**0.0350**
**CD80+/CD86-**	1.786	0.221	9.515	3.403	0.222	9.650	0.555	0.173	4.849	0.1660
**CD80+/CD86+**	0.297	0.200	0.823	0.443	0.159	0.896	0.224	0.198	0.555	0.3930
**CD80+/CD86+**	0.161	0.031	0.614	0.230	0.006	2.604	0.122	0.028	0.307	0.3930
**HLA-DR+/CD4+**	0.519	0.330	2.242	0.932	0.393	2.991	0.411	0.152	0.743	0.1650
**HLA-DR+/CD8+**	0.099	0.055	0.320	0.202	0.086	0.495	0.064	0.040	0.123	0.0520
**HLA-DR+/CD80+/CD86-**	0.211	0.030	0.859	0.571	0.043	1.388	0.055	0.020	0.344	0.0750
**HLA-DR+/CD80-/CD86+**	0.098	0.052	0.188	0.169	0.050	0.228	0.061	0.050	0.111	0.1900
**HLA-DR+/CD80+/CD86+**	0.097	0.027	0.285	0.176	0.006	1.224	0.091	0.024	0.180	**0.0001**
**CD163+**	4.975	1.906	7.608	6.806	1.356	12.832	3.569	1.496	5.854	0.1750
**CD8+**	1.378	0.537	3.870	1.943	0.769	4.704	1.187	0.343	4.233	0.5520
**AE1AE3+/PDL1+**	0.043	0.007	0.327	0.190	0.005	0.386	0.027	0.005	0.074	0.3610
**CD8+/PD1+**	0.274	0.034	0.975	0.304	0.095	0.616	0.115	0.010	1.101	0.3700
**CD8+/PDL1+**	0.013	0.000	0.036	0.016	0.000	0.241	0.011	0.000	0.031	0.5380
**CD8+/OX40+**	0.034	0.006	0.086	0.044	0.020	0.223	0.014	0.005	0.067	0.3820
**CD3+/CD8-**	7.126	3.480	21.449	14.849	4.181	23.654	5.404	3.090	15.897	0.5020
**CD3+/CD8-/PD1+**	0.523	0.195	1.184	0.515	0.249	1.273	0.532	0.106	1.197	0.7100
**CD3+/CD8-/PDL1+**	0.109	0.015	0.255	0.133	0.015	0.824	0.081	0.014	0.260	0.4470
**CD3+/CD8-/FOXP3+**	0.375	0.194	0.662	0.436	0.075	0.609	0.313	0.208	0.682	0.8800
**CD163+/PDL1+**	0.075	0.012	0.319	0.160	0.006	1.272	0.050	0.012	0.167	0.1310
