# Supplementary material for: Prognostic immune markers in esophageal cancer patients managed with trimodal therapy
Source: Cancer Immunol Immunother. 2025 Jan 3;74(2):57. doi: 10.1007/s00262-024-03891-3 (PMC11698998; doi:10.1007/s00262-024-03891-3)
Supplement: Supplementary file 6 [file 262_2024_3891_MOESM7_ESM.docx]

	Overall (n=20)			No relapse (n=9)			Relapse (n=11)			
	Median	1st quartile	3rd quartile	Median	1st quartile	3rd quartile	Median	1st quartile	3rd quartile	p-value
**CD4+**	3.262	1.594	16.952	5.928	1.521	23.397	3.053	1.874	7.929	0.515
**CD8+**	2.211	1.304	5.348	3.316	1.321	7.762	1.900	1.208	3.546	0.515
**HLA-DR+**	1.998	1.049	6.199	4.484	2.226	8.349	1.700	0.840	1.947	**0.0156**
**CD4+/LAG3+**	0.019	0.007	0.078	0.038	0.013	0.165	0.008	0.005	0.050	0.146
**CD4+/TIM3+**	0.084	0.019	0.551	0.135	0.031	4.088	0.029	0.008	0.375	0.23
**CD4+/CTLA4+**	0.129	0.046	0.758	0.457	0.052	2.350	0.111	0.041	0.432	0.315
**CD8+/LAG3+**	0.004	0.000	0.007	0.006	0.004	0.038	0.000	0.000	0.004	**0.0153**
**CD8+/TIM3+**	0.064	0.009	0.159	0.113	0.024	1.681	0.013	0.002	0.104	0.1
**CD8+/CTLA4+**	0.021	0.007	0.044	0.033	0.011	0.121	0.011	0.006	0.026	0.122
**CD80+/CD86-**	1.786	0.295	9.848	5.775	1.259	11.716	1.038	0.220	4.849	0.203
**CD80+/CD86+**	0.246	0.187	0.678	0.467	0.124	1.151	0.224	0.198	0.329	0.408
**CD80+/CD86+**	0.161	0.028	0.721	0.504	0.028	3.903	0.122	0.028	0.318	0.359
**HLA-DR+/CD4+**	0.482	0.275	2.687	1.813	0.398	5.462	0.411	0.167	0.743	0.203
**HLA-DR+/CD8+**	0.088	0.047	0.391	0.251	0.087	0.689	0.058	0.034	0.123	0.122
**HLA-DR+/CD80+/CD86-**	0.211	0.026	1.044	0.788	0.173	1.928	0.055	0.023	0.344	0.068
**HLA-DR+/CD80-/CD86+**	0.082	0.050	0.188	0.159	0.032	0.569	0.061	0.050	0.111	0.274
**HLA-DR+/CD80+/CD86+**	0.091	0.024	0.326	0.227	0.026	1.973	0.079	0.024	0.115	**0.0003**
**CD163+**	5.605	3.138	9.696	10.348	2.331	16.924	3.569	3.138	5.854	0.091
**CD8+**	1.371	0.540	6.319	1.980	0.614	13.665	1.329	0.540	4.233	0.599
**AE1AE3+/PDL1+**	0.038	0.006	0.190	0.114	0.007	0.327	0.027	0.005	0.063	0.482
**CD8+/PD1+**	0.162	0.029	0.596	0.274	0.067	0.331	0.115	0.010	1.101	0.442
**CD8+/PDL1+**	0.011	0.000	0.167	0.018	0.002	1.011	0.009	0.000	0.031	0.334
**CD8+/OX40+**	0.037	0.010	0.067	0.034	0.010	0.099	0.037	0.010	0.067	0.934
**CD3+/CD8-**	6.642	4.425	21.722	17.712	3.244	27.517	5.404	4.425	15.897	0.599
**CD3+/CD8-/PD1+**	0.322	0.098	1.020	0.364	0.103	0.894	0.280	0.098	1.197	1
**CD3+/CD8-/PDL1+**	0.086	0.014	0.565	0.109	0.025	2.219	0.081	0.014	0.260	0.342
**CD3+/CD8-/FOXP3+**	0.313	0.189	0.682	0.285	0.054	0.780	0.313	0.208	0.682	0.657
**CD163+/PDL1+**	0.085	0.012	0.858	0.599	0.026	2.292	0.050	0.012	0.167	0.109
